# A Clinical Prediction Model for Bacterial Coinfection in Children with Respiratory Syncytial Virus Infection: A Development and Validation Study

**DOI:** 10.3390/diagnostics16030403

**Published:** 2026-01-27

**Authors:** Di Lian, Jianxing Wei, Dong Wang, Meiling Xie, Chenye Lin, Qiuyu Tang

**Affiliations:** 1 Fujian Children’s Hospital (Fujian Branch of Shanghai Children’s Medical Center), College of Clinical Medicine for Obstetrics & Gynecology and Pediatrics, Fujian Medical University, Fuzhou 350014, China; liandi@fjmu.edu.cn (D.L.); lcywyyx1@163.com (C.L.); 2 Department of Nephrology, Blood Purification Research Center, The First Affiliated Hospital, Fujian Medical University, Fuzhou 350001, China

**Keywords:** respiratory syncytial virus, bacterial coinfection, prediction model, neutrophil-to-lymphocyte ratio, C-reactive protein, serum amyloid A, nomogram

## Abstract

**Objectives:** Respiratory syncytial virus (RSV) is a leading cause of hospitalization for acute lower respiratory tract infections (ALRIs) in children, with bacterial coinfection complicating diagnosis and often driving antibiotic overuse. This study aimed to develop and validate a clinical prediction model using common laboratory biomarkers to enable early, accurate identification of clinically significant bacterial coinfection in children with RSV infection. **Methods:** A single-center, retrospective cohort study was conducted at Fujian Children’s Hospital, enrolling 518 hospitalized children with RSV infection, which was confirmed via targeted next-generation sequencing (tNGS). Patients were randomly divided into a training set (*n* = 363) and a test set (*n* = 155) at a 7:3 ratio. The primary outcome, bacterial coinfection, was defined by a composite reference standard integrating etiological evidence from tNGS with clinical, inflammatory, and imaging data, and adjudicated by a blinded expert panel. LASSO regression identified independent predictors, followed by multivariable logistic regression modeling. Model performance was assessed via discrimination (AUC), calibration (Hosmer–Lemeshow test), and clinical utility (Decision Curve Analysis) in both sets. **Results:** Neutrophil-to-lymphocyte ratio (NLR), C-reactive protein (CRP), and serum amyloid A (SAA) were selected as predictors. The model achieved an AUC of 0.832 (95% CI: 0.788–0.875) in the training set and 0.811 (95% CI: 0.737–0.885) in the test set, with well-calibrated predictions (*p* > 0.05). Decision curve analysis demonstrated net clinical benefit across 10–80% threshold probabilities. A nomogram was developed for practical application. **Conclusions:** This study established a model integrating NLR, CRP, and SAA. It offers a reliable tool for the early detection of bacterial coinfection in RSV-infected children, enabling targeted antibiotic stewardship and improving clinical outcomes.

## 1. Introduction

Globally, respiratory syncytial virus (RSV) is recognized as the primary pathogen responsible for acute lower respiratory tract infections (ALRIs) among the pediatric population, representing a significant challenge to public health systems [1]. A key challenge in managing RSV infections is the early detection of bacterial coinfection, which exacerbates disease severity and drives antibiotic overuse, particularly when clinical presentations overlap with viral pathology [2,3]. Targeted next-generation sequencing (tNGS) has significantly improved pathogen detection sensitivity. However, distinguishing clinically significant bacterial coinfection from colonization remains challenging, often requiring integrated laboratory approaches [4].

Conventional inflammatory indices, such as C-reactive protein (CRP) and white blood cell count (WBC), lack sufficient diagnostic accuracy for detecting bacterial coinfection in patients with RSV [5]. Emerging evidence suggests that novel inflammatory biomarkers, such as the neutrophil-to-lymphocyte ratio (NLR) and serum amyloid A (SAA), captured through routine laboratory testing, may enhance diagnostic accuracy by reflecting distinct immune responses [6]. However, the diagnostic potential of individual markers is constrained, highlighting the need for multivariable models that leverage laboratory data [7]. The integration of tNGS with conventional biomarkers presents a unique opportunity in laboratory medicine to develop precise diagnostic tools.

The primary objective of this research was to construct and internally validate a predictive tool that integrates NLR, CRP, and SAA for the detection of bacterial coinfection in children with tNGS-verified RSV infection. We further developed a nomogram to visualize this model, providing clinicians with a practical tool to interpret complex laboratory data. This approach aims to support evidence-based antibiotic prescribing and facilitate precision medicine in pediatric RSV cases.

## 2. Materials and Methods

### 2.1. Study Design and Ethical Statement

This single-center, retrospective cohort study was conducted at Fujian Children’s Hospital. The study protocol was approved by the Ethics Committee of Fujian Children’s Hospital (Approval No.: 2025ETKLRK10017) and adhered to the Declaration of Helsinki. Individual informed consent was waived due to the retrospective nature and the use of anonymized data. The development and reporting of this prediction model strictly adhered to the Transparent Reporting of a multivariable prediction model for Individual Prognosis or Diagnosis (TRIPOD) statement [8].

### 2.2. Study Population

We screened all pediatric patients (aged 28 days to 14 years) hospitalized with a primary diagnosis of RSV infection between January 2022 and August 2025. While the initial clinical diagnosis of RSV was established via either multiplex PCR or tNGS, eligibility for this specific analysis was strictly limited to patients with available tNGS results from respiratory tract specimens (nasopharyngeal swabs or bronchoalveolar lavage fluid). To ensure cohort homogeneity, the following exclusion criteria were applied: (1) presence of severe underlying comorbidities that could confound infection assessment (e.g., congenital heart disease, severe immunodeficiency); (2) administration of systemic antibiotics for more than 48 h prior to admission; (3) incomplete electronic medical records regarding key modeling variables; and (4) co-detection of other respiratory viruses (e.g., adenovirus, influenza) or atypical pathogens (e.g., Mycoplasma pneumoniae) via tNGS. This final criterion was implemented to focus the analysis exclusively on distinguishing pure RSV infection from RSV combined with typical bacterial coinfection. Following this selection process, eligible patients were randomly partitioned into a training set and a test set at a 7:3 ratio for model development and internal validation.

### 2.3. Data Collection and Definitions

Data were extracted from the hospital’s electronic medical record (EMR) system by two independent researchers using a standardized form, with discrepancies resolved by consensus. Collected variables included: demographic data (age, sex, weight), clinical outcomes (hospital stay length, severity, ICU admission, mechanical ventilation), imaging findings (chest X-ray/CT: increased markings, consolidation, infiltrates, pleural effusion), and laboratory parameters (first 24 h results: WBC, platelet count, neutrophil/lymphocyte counts, CRP, procalcitonin, SAA, ferritin, LDH, albumin, ALT, AST, D-dimer). Derived indices included NLR and PLR (platelet/lymphocyte ratio). RSV subtyping (A/B) was determined via tNGS.

### 2.4. Targeted Next-Generation Sequencing (tNGS) and Pathogen Identification

Respiratory tract specimens (nasopharyngeal swabs or bronchoalveolar lavage fluid) were collected typically within 24 h of admission and transported to certified third-party clinical laboratories for analysis. Pathogen identification was performed using commercial multiplex PCR-based tNGS assays provided by Dian Diagnostics (Hangzhou, China) or KingMed Diagnostics (Fuzhou/Hangzhou, China). Briefly, total nucleic acids (DNA and RNA) were extracted from 300 µL of the clinical sample using automated extraction systems. For the detection of RNA viruses, reverse transcription was performed to generate cDNA. Library preparation involved multiplex PCR amplification using specific primers designed to target the hypervariable regions or specific gene sequences of pathogens. The constructed libraries were subsequently sequenced on high-throughput platforms, such as the Illumina NextSeq (Illumina, San Diego, CA, USA) or MGISEQ-2000 (MGI Tech, Shenzhen, China). The bioinformatic pipeline included adapter trimming, filtration of low-quality reads and human host sequences, and alignment of high-quality reads against a curated reference database covering over 200 respiratory pathogens. Both assays utilized internal standards to enable semi-quantitative analysis (reported as copies/mL), with a lower limit of detection ranging from 100 to 500 copies/mL.

### 2.5. Outcome Definition and Adjudication

The primary endpoint, clinically significant bacterial coinfection, was determined using a rigorous Composite Reference Standard (CRS) [9]. A patient was classified as coinfected only if tNGS revealed a substantial bacterial load (sequence read count >10,000 or concentration > 10^3^ copies/mL) AND was accompanied by at least two of the following clinical indicators: (1) worsening clinical status (e.g., persistent fever, increased work of breathing); (2) elevated inflammatory biomarkers (procalcitonin > 0.5 µg/L or CRP > 20 mg/L); (3) radiological confirmation of new consolidation or infiltrates; and (4) a positive therapeutic response to targeted antibiotics. While individual indicators may be nonspecific in isolation, their required concurrence (at least two) with a high bacterial load on tNGS creates a highly specific diagnostic profile. To distinguish true pathogenicity from colonization—a critical challenge in respiratory diagnostics—final classification was adjudicated by a blinded panel of two independent pediatricians. Discrepancies were resolved by a senior consultant. Notably, the adjudication process involved triangulation of available microbiological, clinical, inflammatory, and imaging data, specifically accounting for the differences in diagnostic value between specimen types (nasopharyngeal swabs vs. bronchoalveolar lavage fluid) to minimize misclassification bias [10].

### 2.6. Statistical Analysis

The hospital dataset was randomly split into a training set (*n* = 363) and a test set (*n* = 155) at a 7:3 ratio using stratified sampling. Data distribution was assessed with the Shapiro–Wilk test, with normally distributed variables summarized as mean ± standard deviation and non-normally distributed variables as median (interquartile range); baseline comparisons employed Pearson’s chi-squared or Fisher’s exact tests for categorical variables. For continuous data, group comparisons were performed using either Student’s *t*-test or the Mann–Whitney U test, depending on data normality. Records containing missing values (approximately 5.2%) were removed from the final analysis. In the training set, Least Absolute Shrinkage and Selection Operator (LASSO) regression with 10-fold cross-validation (glmnet package in R) identified predictors of bacterial coinfection, followed by multivariable logistic regression modeling. Model performance was evaluated using the area under the receiver operating characteristic curve (AUC, 0.5–1.0), the Hosmer–Lemeshow calibration test, and decision curve analysis (DCA) for net benefit estimation [11,12]. Statistical computations were executed using R software (version 4.2.2, R Foundation for Statistical Computing, Vienna, Austria) and the MSTATA software (https://www.mstata.com/). A two-sided *p*-value of less than 0.05 was considered statistically significant.

## 3. Results

### 3.1. Patient Enrollment and Baseline Characteristics

**Figure 1:**
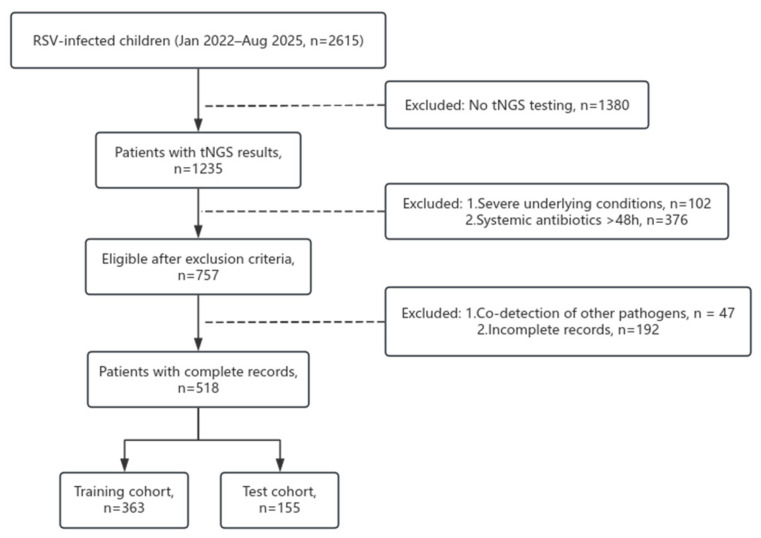
Flow diagram of patient enrollment for the study.

Between January 2022 and August 2025, 2615 children hospitalized with RSV infection were screened at Fujian Children’s Hospital. Of these, 1235 underwent targeted next-generation sequencing (tNGS), with 102 excluded due to severe underlying conditions (e.g., congenital heart disease), 376 due to systemic antibiotic use >48 h pre-admission, 47 due to co-detection of other respiratory pathogens (e.g., adenovirus, influenza), and 192 due to incomplete records, yielding 518 eligible patients. These were randomly allocated to a training set (*n* = 363) and a test set (*n* = 155) at a 7:3 ratio (Figure 1). Baseline characteristics showed no significant differences between cohorts in sex (male: 57.9% vs. 60.6%, *p* = 0.554), median age (12 vs. 12 months, *p* = 0.271), or weight (10.7 vs. 9.9 kg, *p* = 0.325), with laboratory and clinical features also balanced (all *p* > 0.05, Table 1), confirming successful randomization.

### 3.2. Univariate Analysis of Risk Factors for Bacterial Coinfection

In the training set (*n* = 363), 129 patients (35.5%) were adjudicated with bacterial coinfection. Univariate analysis revealed significant differences: median age (24 vs. 12 months, *p* < 0.001), white blood cell count (10.2 vs. 8.2 × 10^9^/L, *p* < 0.001), NLR (1.64 vs. 0.54, *p* < 0.001), PLR (103 vs. 74, *p* < 0.001), CRP (11 vs. 3 mg/L, *p* < 0.001), procalcitonin (0.12 vs. 0.09 µg/L, *p* < 0.001), and SAA (52 vs. 20 mg/L, *p* < 0.001) were higher in the coinfection group (Table 2), suggesting these markers’ potential in distinguishing infection states.

### 3.3. Development of the Predictive Model via LASSO Regression

**Figure 2:**
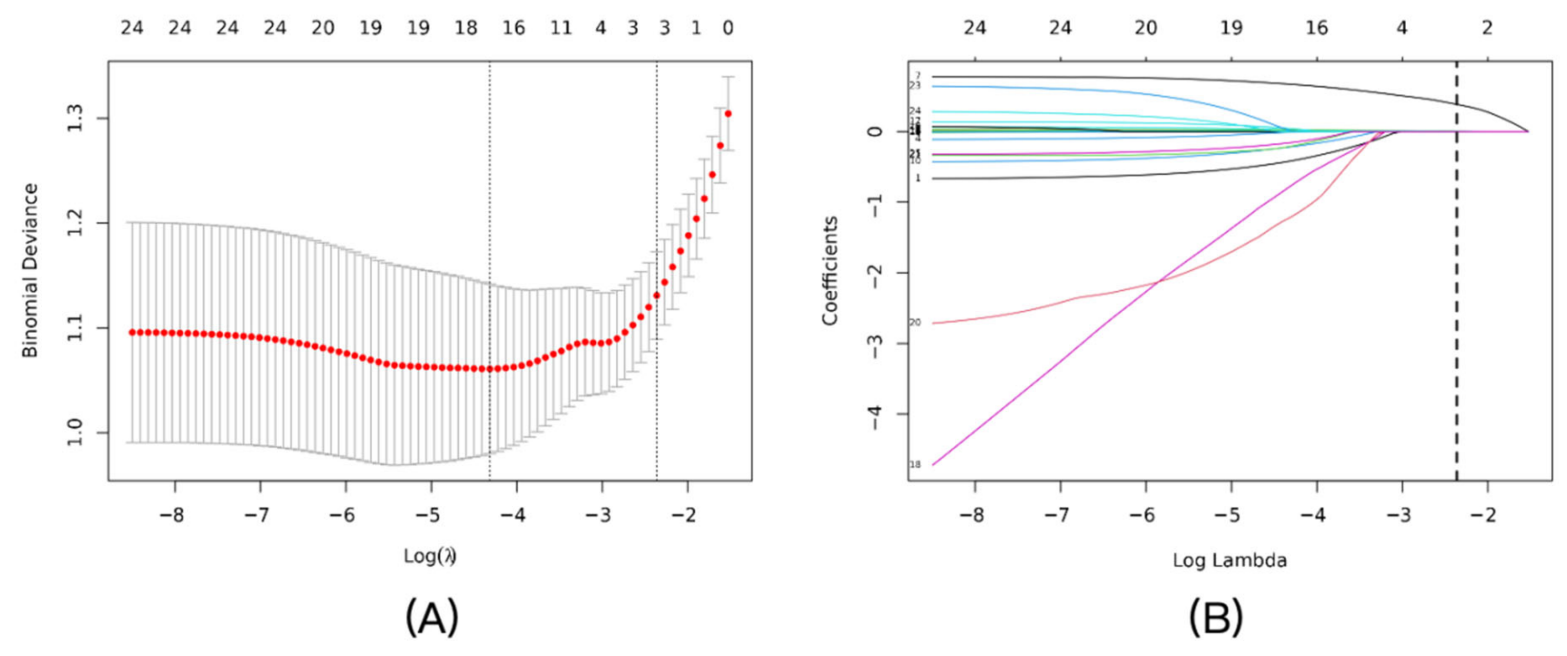
Feature selection using the LASSO logistic regression model: (**A**) Tuning parameter (λ) selection in the LASSO model using 10-fold cross-validation. The binomial deviance was plotted versus log(λ). The vertical dotted lines indicate the optimal λ value (λ_min, left line) that results in the minimum mean cross-validated error, and the λ_1se value (right line) that corresponds to the most regularized model within one standard error of the minimum. We chose λ_1se to build the most parsimonious model. (**B**) LASSO coefficient profiles of the candidate predictors. A vertical line is drawn at the optimal λ value selected by the cross-validation process, where three features remained with non-zero coefficients.

LASSO regression with 10-fold cross-validation in the training set identified NLR, CRP, and SAA as key predictors, with an optimal penalty coefficient λ = 0.0943 (Figure 2A). The coefficient profile plot showed variable shrinkage, retaining these three markers (Figure 2B). Multivariable logistic regression confirmed their independence: NLR (OR = 2.13, 95% CI: 1.64–2.79, *p* < 0.001), CRP (OR = 1.03, 95% CI: 1.01–1.06, *p* = 0.017), and SAA (OR = 1.01, 95% CI: 1.00–1.01, *p* = 0.007) (Table 3).

### 3.4. Nomogram for Clinical Application

**Figure 3:**
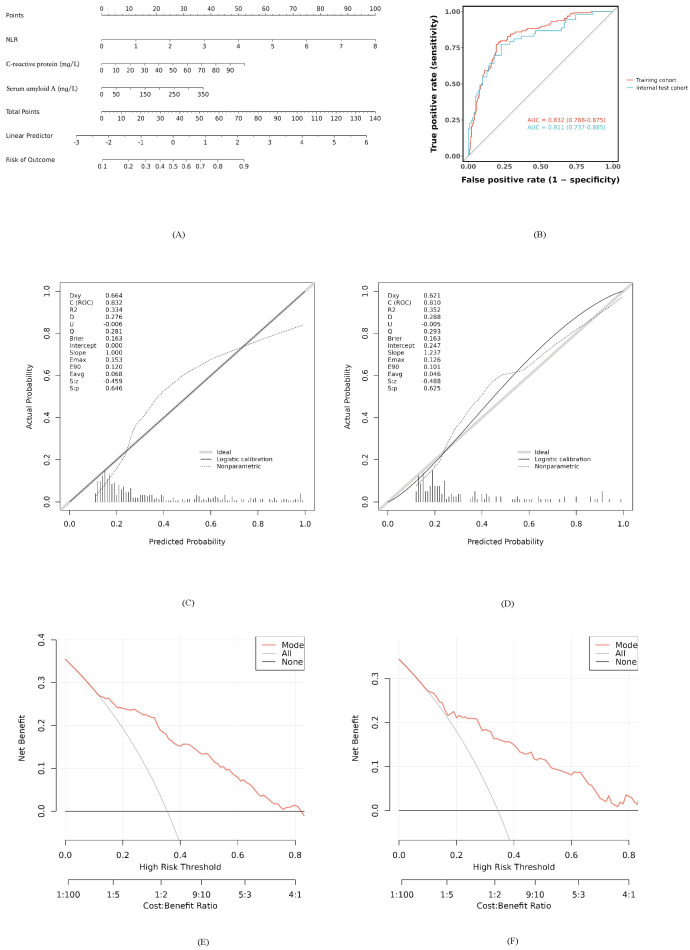
Development and performance of the nomogram for predicting bacterial coinfection: (**A**) The nomogram developed based on the three independent predictors (NLR, CRP, and SAA) in the training cohort. (**B**) ROC curves illustrating the discriminative performance of the nomogram across both the training and test cohorts. (**C**) Calibration plot for the training set, where the y-axis depicts observed probabilities and the x-axis shows predicted probabilities; the dashed diagonal line indicates ideal prediction. (**D**) Calibration assessment for the test cohort. (**E**) Clinical utility evaluation via decision curve analysis (DCA) in the training set, with the y-axis denoting net benefit. The red solid line represents the nomogram. The thin gray line represents the “treat-all” strategy, and the black horizontal line represents the “treat-none” strategy. (**F**) Decision curve analysis (DCA) for the nomogram in the test cohort.

Based on the developed model, a nomogram was constructed to estimate bacterial coinfection probability, integrating NLR, CRP, and SAA values. Clinicians can sum points from each variable’s axis and project the total onto a risk scale for rapid assessment (Figure 3A).

### 3.5. Performance and Validation of the Predictive Model

The model’s discriminative ability was strong, with an AUC of 0.832 (95% CI: 0.788–0.875) in the training set and 0.811 (95% CI: 0.737–0.885) in the test set (Figure 3B). Calibration curves showed good agreement between predicted and observed probabilities (Hosmer-Lemeshow *p* > 0.05, Figure 3C,D). Decision curve analysis indicated net clinical benefit across 10–80% threshold probabilities (Figure 3E,F), supporting practical utility.

### 3.6. Pathogen Distribution of Bacterial Coinfections

Among 129 patients with bacterial coinfection, tNGS identified *Haemophilus influenzae* (98 cases, 18.92%), *Streptococcus pneumoniae* (65 cases, 12.55%), *Moraxella catarrhalis* (28 cases, 5.41%), *Bordetella pertussis* (12 cases, 2.32%), *Staphylococcus aureus* (10 cases, 1.93%), and *Klebsiella pneumoniae* (9 cases, 1.74%) as predominant pathogens (Table 4).

## 4. Discussion

In this study, we successfully developed and validated a clinical prediction model that integrates the NLR, CRP, and SAA for the early identification of clinically significant bacterial coinfection in children hospitalized with RSV infection. The model demonstrated not only excellent discrimination (AUC > 0.8) and good calibration in internal validation but, more importantly, its practical utility was confirmed by decision curve analysis across a wide range of clinical thresholds. To our knowledge, this is one of the first studies to integrate these three common inflammatory markers for this specific clinical scenario, providing a novel, evidence-based tool to address a persistent diagnostic challenge and promote precision antibiotic stewardship in pediatrics.

Our multivariable analysis revealed that NLR, CRP, and SAA were all independent predictors of bacterial coinfection in children with RSV-ALRI. As a marker of systemic inflammation, an elevated NLR reflects an immune status characterized by neutrophil activation and lymphocyte suppression, which is closely associated with the host’s stress response during bacterial infection [13,14]. The odds ratio for NLR in our study was 2.13 (95% CI: 1.64–2.79), indicating that for each unit increase in NLR, the risk of bacterial coinfection approximately doubles. This finding is consistent with numerous studies in sepsis and intra-abdominal infections, where NLR has been proven to be a crucial indicator for predicting infection severity and prognosis [15]. As an acute-phase protein, CRP levels rise significantly during bacterial infections; although the OR in our study was modest at 1.03 (95% CI: 1.01–1.06), its dynamic changes should not be overlooked as an indicator of bacterial infection [16]. SAA, another sensitive inflammatory marker, had an OR of 1.01 (95% CI: 1.00–1.01), further supporting its value in differentiating viral from bacterial infections. The combined application of these inflammatory markers, integrated through a multivariable model, significantly improves the predictive accuracy for bacterial coinfection, overcoming the limitations of any single marker.

Compared to the existing literature, the significant innovation of our study lies in its methodological rigor and specific clinical focus. First, regarding outcome definition, we addressed the difficulty of distinguishing true pathogens from colonizers in tNGS results. Rather than relying solely on positive tNGS reads, we applied a composite reference standard. Instead, we adopted a composite reference standard encompassing etiological, clinical, inflammatory, and imaging evidence, adjudicated through a blinded expert panel process [17]. This approach minimizes misclassification bias of the outcome event, ensuring that our model predicts a “clinically significant” infection that truly warrants intervention, rather than asymptomatic carriage. This greatly enhances the clinical relevance of our findings. Second, in terms of statistical strategy, the application of LASSO regression not only resolved the issues of subjectivity and collinearity in traditional multivariable analysis but also constructed a data-driven, parsimonious model with only three core predictors [18]. This simplicity is key to the model’s potential for clinical translation, as it is easy to remember, calculate, and implement. The robustness of this statistical approach was particularly evident in addressing potential confounders. Although patients in the coinfection group were older (median 24 vs. 12 months), our LASSO regression analysis effectively controlled for this. Age was included as a candidate variable alongside inflammatory markers. However, the algorithm did not select Age, but instead retained NLR, CRP, and SAA as the most powerful predictors. This indicates that the elevated inflammatory markers are independent indicators of bacterial coinfection rather than a proxy for age-related immune responses.

This study also provides important local microbiological evidence for clinical practice. We found that *Haemophilus influenzae* and *Streptococcus pneumoniae* are the predominant pathogens in children with RSV and bacterial coinfection. This finding is consistent with the pathogen spectrum of community-acquired pneumonia in children in many regions, but clarifying their leading role in the context of RSV infection provides more precise guidance for empirical antibiotic selection [19,20]. For example, while awaiting etiological results, choosing an antibiotic that effectively covers these two pathogens (e.g., amoxicillin–clavulanate or a second/third-generation cephalosporin) would be a more evidence-based decision for RSV-infected children with a high suspicion of bacterial coinfection [21,22]. The efficacy of such targeted treatment was clearly reflected in our cohort’s data. Interestingly, our univariate analysis showed no significant difference in the length of hospital stay or the rate of severe disease between the coinfection and non-coinfection groups. This finding likely reflects the “treatment paradox” often observed in retrospective studies. In our cohort, 100% of the patients in the bacterial coinfection group received targeted antibiotic therapy (predominantly the regimens mentioned above) based on clinical judgment or tNGS results. This timely intervention effectively mitigated disease progression, resulting in clinical outcomes comparable to those of the viral-only group. This underscores the clinical value of our prediction model: without early identification and subsequent antibiotic treatment, these high-risk patients would likely have experienced worse outcomes.

Despite its strengths, our study has limitations. As a single-center, retrospective analysis, our findings need to be interpreted with caution, as patient demographics and local practice patterns may have influenced the results. The model’s generalizability, therefore, remains to be established through external validation. While our sample size was sufficient for the primary analysis, it may not have been robust enough for detailed subgroup evaluations, such as across different age brackets. Additionally, although we included Bordetella pertussis in our coinfection analysis due to its clinical significance, we acknowledge that it represents a distinct pathology compared to typical bacterial superinfections. Furthermore, our model was intentionally parsimonious, relying on three common inflammatory markers; future iterations could potentially be enhanced by incorporating other variables, like viral load or additional cytokines. To further facilitate clinical utility, future work will focus on integrating the prediction formula directly into EMR systems, allowing for automated risk calculation to complement the manual nomogram.

These limitations naturally guide our future work. An external, multicenter prospective validation is the immediate and essential next step to confirm the model’s robustness across diverse populations. Future studies could also explore dynamic models that track biomarker changes over the first 48 h to further improve predictive accuracy. Ultimately, the true clinical value of this tool can only be confirmed through a randomized controlled trial (RCT) designed to test whether a model-guided antibiotic strategy improves patient outcomes, such as reducing antibiotic usage and hospital stay.

## 5. Conclusions

In conclusion, this study successfully developed and validated a clinical prediction model integrating NLR, CRP, and SAA. This model functions as a straightforward and objective instrument to assist healthcare providers in the timely and precise detection of vulnerable patients with clinically significant bacterial coinfection in the complex clinical scenario of RSV infection. It therefore provides valuable insights for achieving precision antibiotic therapy and enhancing antimicrobial stewardship (AMS). However, given the retrospective nature of this study, external validation is required before widespread clinical implementation.

## Figures and Tables

**Table 1:** Baseline characteristics of patients in the training and test cohorts.

Characteristic	Training Cohort (*n* = 363)	Test Cohort (*n* = 155)	*p*-Value
**Demographics**			
Sex, *n* (%)			0.554 ^1^
Female	153 (42.1)	61 (39.4)	
Male	210 (57.9)	94 (60.6)	
Age, months, median (IQR)	12 (7, 24)	12 (6, 24)	0.271 ^2^
Weight, kg, median (IQR)	10.7 (8.0, 13.8)	9.9 (7.8, 13.3)	0.325 ^2^
**Clinical Outcomes**			
Length of stay, days, median (IQR)	6.0 (5.0, 7.0)	5.0 (5.0, 7.0)	0.579 ^2^
Severe disease, *n* (%)	54 (14.9)	23 (14.8)	0.991 ^1^
ICU admission, *n* (%)	5 (1.4)	6 (3.9)	0.094 ^3^
Mechanical ventilation, *n* (%)	4 (1.1)	3 (1.9)	0.432 ^3^
**Laboratory Parameters, median (IQR)**			
WBC (×10^9^/L)	8.8 (6.6, 12.3)	9.1 (6.9, 11.6)	0.517 ^2^
Platelet count (×10^9^/L)	332 (260, 425)	334 (271, 424)	0.584 ^2^
NLR	0.79 (0.42, 1.59)	0.77 (0.48, 1.29)	0.557 ^2^
PLR	81 (58, 116)	77 (56, 113)	0.373 ^2^
CRP (mg/L)	4 (2, 12)	5 (2, 14)	0.552 ^2^
Procalcitonin (μg/L)	0.09 (0.06, 0.18)	0.09 (0.06, 0.17)	0.477 ^2^
SAA (mg/L)	28 (17, 63)	32 (18, 65)	0.652 ^2^
Ferritin (μg/L)	148 (99, 246)	145 (94, 248)	0.857 ^2^
Lactate dehydrogenase (U/L)	323 (283, 373)	324 (276, 383)	0.917 ^2^
Albumin (g/L)	44.9 (42.7, 46.4)	45.2 (42.8, 47.0)	0.228 ^2^
ALT (U/L)	19 (15, 28)	20 (16, 27)	0.568 ^2^
AST (U/L)	42 (35, 50)	42 (35, 50)	0.713 ^2^
D-dimer (mg/L)	0.39 (0.28, 0.52)	0.36 (0.26, 0.48)	0.330 ^2^
**Imaging Findings, *n* (%)**			
Increased lung markings	112 (30.9)	51 (32.9)	0.646 ^1^
Consolidation	19 (5.2)	10 (6.5)	0.581 ^1^
Patchy infiltrates	77 (21.2)	29 (18.7)	0.518 ^1^
Pleural effusion	0 (0.0)	1 (0.6)	0.299 ^3^
**RSV Subtype, *n* (%)**			
RSV-A	106 (29.2)	44 (28.4)	0.852 ^1^
RSV-B	256 (70.5)	111 (71.6)	0.803 ^1^

Data are presented as *n* (%) for categorical variables and median (interquartile range, IQR) for continuous variables. Abbreviations: WBC, white blood cell count; CRP, C-reactive protein; SAA, Serum amyloid A; ALT, alanine aminotransferase; AST, aspartate aminotransferase; ICU, intensive care unit; IQR, interquartile range; NLR, neutrophil-to-lymphocyte ratio; PLR, platelet-to-lymphocyte ratio; RSV, respiratory syncytial virus. ^1^ Pearson’s chi-squared test. ^2^ Wilcoxon rank-sum test. ^3^ Fisher’s exact test.

**Table 2:** Univariate analysis of risk factors for bacterial coinfection in the training cohort (*n* = 363).

Characteristic	No Bacterial Coinfection (*n* = 234)	Bacterial Coinfection (*n* = 129)	*p*-Value
**Demographics**			
Sex, *n* (%)			0.055 ^1^
Female	90 (38.5)	63 (48.8)	
Male	144 (61.5)	66 (51.2)	
Age, months, median (IQR)	12 (5, 24)	24 (11, 36)	<0.001 ^2^
Weight, kg, median (IQR)	10.0 (7.6, 13.0)	12.0 (9.0, 15.5)	<0.001 ^2^
**Clinical Outcomes**			
Length of stay, days, median (IQR)	6.0 (5.0, 7.0)	6.0 (5.0, 7.0)	0.974 ^2^
Severe disease, *n* (%)	35 (15.0)	19 (14.7)	0.953 ^1^
ICU admission, *n* (%)	5 (2.1)	0 (0.0)	0.165 ^3^
Mechanical ventilation, *n* (%)	4 (1.7)	0 (0.0)	0.302 ^3^
**Laboratory Parameters, median (IQR)**			
WBC (×10^9^/L)	8.2 (6.3, 11.2)	10.2 (7.6, 13.4)	<0.001 ^2^
Platelet count (×10^9^/L)	333 (263, 413)	324 (258, 436)	0.455 ^2^
NLR	0.54 (0.33, 1.00)	1.64 (0.95, 2.85)	<0.001 ^2^
PLR	74 (53, 100)	103 (74, 151)	<0.001 ^2^
CRP (mg/L)	3 (2, 7)	11 (4, 22)	<0.001 ^2^
Procalcitonin (μg/L)	0.09 (0.06, 0.14)	0.12 (0.07, 0.27)	<0.001 ^2^
SAA (mg/L)	20 (15, 42)	52 (22, 119)	<0.001 ^2^
Ferritin (μg/L)	144 (95, 246)	150 (112, 220)	0.537 ^2^
Lactate dehydrogenase (U/L)	325 (290, 390)	307 (272, 350)	0.002 ^2^
Albumin (g/L)	45.0 (43.0, 46.8)	44.2 (42.3, 46.0)	0.133 ^2^
ALT (U/L)	20 (16, 30)	18 (15, 24)	0.013 ^2^
AST (U/L)	43 (36, 52)	39 (31, 46)	<0.001 ^2^
D-dimer (mg/L)	0.39 (0.27, 0.50)	0.40 (0.29, 0.54)	0.234 ^2^
**Imaging Findings, *n* (%)**			
Increased lung markings	78 (33.3)	34 (26.4)	0.168 ^1^
Consolidation	11 (4.7)	8 (6.2)	0.539 ^1^
Patchy infiltrates	48 (20.5)	29 (22.5)	0.661 ^1^
Pleural effusion	0 (0.0)	0 (0.0)	>0.999 ^3^
**RSV Subtype, *n* (%)**			
RSV-A	75 (32.1)	31 (24.0)	0.108 ^1^
RSV-B	159 (67.9)	97 (75.2)	0.147 ^1^

Data are presented as *n* (%) for categorical variables and median (interquartile range, IQR) for continuous variables. Variables with statistically significant differences (*p* < 0.05) are highlighted in bold. Abbreviations: WBC, white blood cell count; CRP, C-reactive protein; SAA, serum amyloid A; ALT, alanine aminotransferase; AST, aspartate aminotransferase; ICU, intensive care unit; IQR, interquartile range; NLR, neutrophil-to-lymphocyte ratio; PLR, platelet-to-lymphocyte ratio; RSV, respiratory syncytial virus. ^1^ Pearson’s chi-squared test. ^2^ Wilcoxon rank-sum test. ^3^ Fisher’s exact test.

**Table 3:** Multivariable logistic regression analysis of independent predictors for bacterial coinfection in the training cohort.

Characteristic	β (Beta)	SE	OR (95% CI)	*p*-Value
NLR	0.758	0.136	2.13 (1.64–2.79)	<0.001
C-reactive protein (mg/L)	0.032	0.013	1.03 (1.01–1.06)	0.017
Serum amyloid A (mg/L)	0.006	0.002	1.01 (1.00–1.01)	0.007

Abbreviations: β, beta coefficient; SE, standard error; OR, odds ratio; CI, confidence interval; NLR, neutrophil-to-lymphocyte ratio.

**Table 4:** Distribution and classification of bacterial pathogens identified in children with RSV coinfection.

Pathogen	Type	Number of Detections (*n*)	Detection Rate (%) ^1^
*Haemophilus influenzae*	G−	98	18.92
*Streptococcus pneumoniae*	G+	65	12.55
*Moraxella catarrhalis*	G−	28	5.41
*Bordetella pertussis*	G−	12	2.32
*Staphylococcus aureus*	G+	10	1.93
*Klebsiella pneumoniae*	G−	9	1.74
*Pseudomonas aeruginosa*	G−	2	0.39
*Streptococcus intermedius*	G+	2	0.39
*Streptococcus pyogenes*	G+	1	0.19

Abbreviations: G−, Gram-negative; G+, Gram-positive. ^1^ The detection rate was calculated based on the total study population (*n* = 518).

## Data Availability

The data presented in this study are available upon request from the corresponding author. The data are not publicly available due to privacy and ethical restrictions.
